# Midwifery students’ experiences of learning clinical skills in Iran: a qualitative study

**DOI:** 10.5116/ijme.5a88.0344

**Published:** 2018-03-09

**Authors:** Golnoosh Ahmadi, Mohsen Shahriari, Mahmood Keyvanara, Shahnaz Kohan

**Affiliations:** 1 Student Research Committee, School of Nursing and Midwifery, Isfahan University of Medical Sciences, Isfahan, Iran; 2 Nursing and Midwifery Care Research Center, Adult Health Nursing, Isfahan University of Medical Sciences, Isfahan, Iran; 3 Social Determinants of Health Research Center, Isfahan University of Medical Sciences, Isfahan, Iran; 4 Nursing and Midwifery Care Research Center, Faculty of Nursing and Midwifery, Isfahan University of Medical Sciences, Isfahan, Iran

**Keywords:** Midwifery students, learning, clinical skills, students’ experiences, Iran

## Abstract

**Objective:**

To explore how midwifery students in Iran experience learning clinical skills.

**Methods:**

A qualitative study was used. Midwifery students from three universities in Iran participated. The study used a convenience sample of eighteen students. Data for this study was collected using semi-structured interviews (N=12) and focus groups (N=6). Data were recorded on a digital audio recorder and then transcribed. The qualitative data were analyzed using a content analysis approach.

**Results:**

Six broad themes emerged from the analysis: Limited opportunities to experience skills, difficulties with course plan gaps, need for creating a supportive clinical environment, learning drives, confusion between different methods, and stress in the clinical setting. Short verbatim quotations from the participants were presented to provide evidence for the interpretation of data.

**Conclusions:**

The findings of this study have provided a clear picture of the factors and mechanisms involved in learning clinical skills by midwifery students. This study showed that students had some difficulties and concerns during learning of clinical midwifery skills. The findings of this study suggest that midwifery educators conduct further studies to tackle these issues in clinical skills learning. The findings of this study are subject to some limitations which are discussed.

## Introduction

Midwifery education varies across the world. In Iran, it is a four-year undergraduate program. The admission process for studying undergraduate midwifery is based on a competitive national examination. The higher the rank of the exam, the more chance of being offered a place on the course of midwifery. Approximately all of the midwifery students are school leavers, and they are not experienced in hospital environments.

Ministry of Health and Medical Education has designed the undergraduate midwifery curriculum for all universities across the country. A substantial amount of the course is allocated a variety of clinical skills during placement. After the first semester, students enter clinical settings in groups of 4 to 8 members under the supervision of clinical instructors. These are faculty full- or part-time employees and are not hospital midwives. To complete the course, the students should be the main birth attendant in at least 80 normal births, and then successfully pass the final clinical exam indicating that the students demonstrate their competency in managing different patients in clinical situations.

The provision of maternity service in labor wards of Iran is mostly organized by the medical model of care, and the midwives work under the supervision of obstetricians. There are few cases of the midwifery-led model in which autonomous midwives attend the births of their clients.

The undergraduate midwifery course aims to equip the students with the practical skills necessary to become practicing professional midwives.^1^^,^^2^ Clinical skills underpin midwives’ professional practice, and therefore students should have an opportunity to learn, develop and master clinical skills.^3^

To provide these opportunities, understanding the teaching and learning process of midwifery students will enable midwifery educators to enhance clinical competencies. Therefore, there is an international call for exploring midwifery students’ experiences of learning clinical skills.^4^^,^^5^ Indeed, very little was found during the literature review of this topic.

Some studies showed different aspects of student midwives’ learning experiences, however. For example, in a study, midwifery students stated that midwives who train the students needed to be updated about the teaching and learning strategies in clinical settings.^6^  In another study in Australia, students perceived achievement of competency standards and confidence to practice difficult because of the restricted nature of midwifery practice within hospitals in which they were learning.^7^  Furthermore, the results of a study in England showed the importance of providing adequate support and feedback by educators and mentors to promote the transfer of knowledge and skills into the workplace.^8^ Further research has also shown that both the midwives and the clinical settings might generate educational sources of stress in midwifery students.^9^

In Iran, achievement of the competencies for student midwives has been studied. The findings of these studies show that midwifery skills in today students have declined in comparison with the last generation students.^10^^-^^13^ Lead midwives for education should take into account these findings and carry out further research to improve the quality of clinical skills of midwifery students in Iran.

In Iran, there are also a number of surveys about midwifery students’ perspective on the current status of clinical education^14^^,^^15^ clinical education problems,^16^ stressors,^17^ students’ satisfaction with clinical education,^18^ perceived feedback^19^ as well as support and supervision^20^ in the clinical environment. All of these studies were conducted using quantitative approaches, so they did not provide a rich description of the learning process of midwifery students. To our knowledge, only one phenomenological study was conducted in Iran focusing on the experiences of midwifery graduates about clinical learning. In this study, the researchers concluded that instructor performance, pre-clinical training, and students’ satisfaction are the key factors associated with clinical skills learning. Lack of peripheral facilities, along with lack of coordination of educational planning, as well as behaviors of health care personnel are inhibiting factors for learning clinical skills.^21^

These findings show that there has been little qualitative analysis of clinical skills learning in midwifery students and much uncertainty still exists about midwifery education in Iran. Consequently, providing a rich and thick description of the experience of students using a qualitative inquiry approach provides readers with a proxy experience for improving the quality of midwifery education in Iran. Hence, the objective of this investigation is to explore the experience of midwifery students in the context of learning clinical skills. This study also aimed to address the following research question: how midwifery students experience clinical skills learning in Iran?

## Methods

### Research design

The methodological approach taken in this study is a qualitative inquiry approach. By employing this approach, we attempted to provide an in-depth description of the experience of midwifery students with regard to learning clinical skills.

### Setting and participants

A multi-center qualitative study was conducted in three universities: Tehran University of Medical Sciences (TUMS), Shahid Beheshti University of Medical Sciences (SBUMS), and Isfahan University of Medical Sciences (IUMS). The participants in this study were midwifery students. Prior to undertaking the investigation, ethical approval was obtained from the ethics committee of Isfahan University of Medical Sciences. With regards to the ethical considerations, the researchers elaborated the aim of the study, who will conduct the interviews, and how the data will be used. In addition, the students understood that their participation was entirely voluntary, and their responses were anonymous. All responses were also kept confidential. What is more, the students were aware that they could easily withdraw from the study at any time and without fear of retribution. Before the research proceed, the written consent forms were signed off by the res students to indicate consent.

As regards informing participants, lecturers explained the purpose of the study for the students and then invited them to take part in the study. The lecturers received the contact numbers from those students who agreed to take part in the study. Next, GA contacted the students to arrange a convenient time and place for the interview.

In this study, eighteen undergraduate midwifery students agreed to participate in an individual interview and a focus group. The students were between the ages of 2 and 30. Table 1 shows some of the main characteristics of the students.

**Table 1:** The main characteristics of the students who participated in an individual interview and a focus group

University	Number of participants in interviews	Number of participants in focus group
IUMS	S3=1, S5=1, S7=1, S8=2	Year 4= 2
SBUMS	S4=1, S7=1, S8=1	Year 4= 2
TUMS	S6=2, S7=1, S8=1	Year 4= 2

Key: S: Semester, e.g., S3 should read Semester 3

### Data collection

For this study, qualitative data were collected using convenience sampling. Semi-structured interviews conducted with 12 students to collect focused, qualitative textual data. In addition, one focus group discussion was conducted with six final year midwifery students was directed. The purpose of the focus group discussion was to reveal dynamics and issues, and, also for cross-checking and triangulation the qualitative data from different sources which in turn enhance the validity of the study.^22^ Additionally, the focus group discussion confirmed data saturation.

To conduct interviews, an interview guide was developed to ensure that the interviewer collects similar types of data from all students^.22^ A panel of experts established the content validity of the interview guide.

The interviews were held at times and places that were convenient to each participant. Examples of interview questions to elicit the students’ learning experience of clinical skills learning were: ‘Would you tell me about your experiences of clinical skills learning in midwifery?’, ‘Which factors helped you to learn the clinical skills better?’, ‘Would you please tell me about your relationship with others (for example midwives, instructors, …) in a clinical setting?’ The interviewer probed the student responses by using questions or statements, such as ‘could you tell me something more about that?’, ‘could you give me an example’ and ‘what you mean by?’

In the focus group discussion, the students were encouraged to talk to one another, ask questions, exchange anecdotes and comments towards each other’s experiences and perspectives. Each interview lasted between 40 and 60 minutes, an average of 50 minutes. The focus group discussion lasted 85 minutes. Each of the interviews was digitally recorded and then fully transcribed. Interviews were conducted in the Persian language. Using the back-translation process, the data were translated into English for this paper.

### Data extraction and analysis

As we were interested in exploring the experience from the views of the study participants, we followed the following steps to conduct a content analysis approach.^23^

In the first step, the interview transcripts were re-read carefully to gain a deeper understanding of the qualitative data. In the second step, interview transcripts were split into small meaningful units. In the third step, the small meaningful units generated categories by bringing several codes together. In the final step of data analysis, categories were reviewed for emerging themes and labelling them. GA conducted these steps manually. However, the research team was also involved in the steps of the analysis process. It should also be emphasized that before the data were submitted to content analysis, the interview transcripts were sent to the students to approve. All students were satisfied with the interview transcripts.

## Results

Six broad themes were generated from the interview transcripts. They are: 1) limited opportunities to experience skills, 2) difficulties with the course plan gaps, 3) need for creating a supportive clinical environment learning, 4) learning drives, 5) confusion between different methods, And 6) stress in the clinical setting. Short verbatim quotations from the participants are presented to provide evidence for the interpretation of data.

### Limited opportunities to experience skills

Most of the participants expressed great concerns about accessing clinical learning opportunities. Because of the high number of students in clinical placements groups, they sometimes had to wait a considerable time for their turn for clinical experience. As a result, some students entered the fourth year of education without sufficient competency even in carrying out the primary tasks such as establishing the intravenous access. In these situations, the students’ confidence decreased, and they became worried about that they could not achieve the minimum midwifery experiences before the end of the course. One student commented:

“I only managed two births by semester seven and actually in both of them my instructor did the main tasks. In the first clerkship experience in semester seven, I managed a birth very clumsily. My instructor shouted: aren’t you a final year student?!” (Semester 8 student)

To achieve 80 required vaginal births within the timeframe of the educational program, some universities arranged fourth-year students to undertake their placements in different non-educational hospitals which had high birth rates. These hospitals were not student-oriented and mostly regarded the students as workforce than learners. At these hospitals, students managed births without the supervision of an instructor, wishing to get help from staff. However, most of the staff were not eager to accept the responsibility of teaching students due to their heavy workload. The students frequently stated that the personnel made them do ward chores rather than attend the birth. These poor educational environments made participants frustrated and distressed. Besides, there were limited learning outcomes for them in these situations. One student stated that:

“After working nonstop and doing several ward tasks, when it is the time of birth, they [midwives] push you aside. This is really unfair. How should we learn to manage a birth then?” (Semester7 student)

Other difficulties centered around competition, and, in some cases conflicts between midwifery students and obstetric residents, who also needed experience conducting births. Another student reflected:

“You look after the birthing woman a long time. Finally, the woman comes to 10 cm and ready to birth and a second-year resident comes and says: this birth is for our first-year resident […] they [residents] do not even see the right of learning for us.”

### Difficulties with course plan gaps

In the universities of the study, theoretical and practical parts of each study units were provided simultaneously in one semester. It became problematic when the students, particularly at the beginning of the semesters, entered clinical fields while they had not learned the prerequisite related theoretical knowledge yet. Participants felt that in these situations their optimal learning was hindered:

“We entered the labor ward while we didn’t know anything about birth or care of birthing women or suchlike things... it was just like we were on another planet…” (Semester 4 student)

According to the curriculum of the undergraduate midwifery course, there are not any obstetrics study units for provision in semester six. During this gap, students identified that their previously learned skills and knowledge diminished. They found it difficult and time-consuming to retrieve the previous level of competency at the beginning of the semester seven. For example, one student pointed out:

“Semester 7 was a great challenge for us. We didn’t have any labor ward placement all over semester 6. It is really long time. We had forgotten everything that we had learned before.” (Semester 8 student)

### Need for creating a supportive clinical environment learning

The supportive clinical environment encompassed receiving support from their clinical instructors and the staff for our participants. The study participants regarded instructors supportive if they trusted the students and provided them opportunities to experience while we are ready to support in case of a need:

“You know? Some instructors put a lot of stress on us and hurry us…some of them don’t let us do the tasks at all. On the other hand, we had a nice instructor. She looked after you calmly while pushed you to carry out the tasks.” (Semester 5 student)

The participants identified that a trainer could be supported only if she were confident about her scientific and practical capabilities.

"Trainers give freedom to the student to do clinical tasks, only when they are self-confident and know that they can fix things if the student messed up." (Semester 5 student)

Since the clinical training took place in the territory of midwives, their cooperation and support were very important in this respect. In the view of our participants, the supportive manner of staff meant being patient with the students when they were carrying out the procedures and not putting pressure on them to hurry up.  Although there were limited numbers of staff who were supportive of this study, their behavior was inhibiting for clinical learning in most of the cases:

"Some midwives deal with the students as if they have never been student themselves. When a procedure takes a little bit longer, they lose their patience and begin to nag." (Semester 7 student)

### Learning drives

There were numbers of motives that pushed the students to learn. First, clinical midwifery practice seemed attractive for some of them. They chased the minutes to attend the birth. As their competency expanded and they learned new skills, they became encouraged to experience more and more skills:

"I love to manage a birth. It is wonderful when you help a birthing mother… I love that when I catch the baby and when I am witnessing the happiness and emotion of the mother just after birth. As I progressed with my midwifery practice skills, I felt a strong enthusiasm to experience more." (Semester 8 student)

Moreover, the participants recognized the necessity of obtaining the minimum midwifery skills to pass the final clinical exam and afterward, practicing as a midwife. This obligation urged the participants for learning skills:

"I'm always worried about how I can pass the final exam if I do not learn the skills? The time is running, and I feel there are lots of tasks that I have not learned yet… I must try more…" (Semester 5 student)

### Confusion between different methods

Nearly all of the participants stated there were times that they felt confused about what is really true. One of the causes of confusion was the gap between theory and practice. This means that students' expectations, created by the theoretical education in the university, did not match the situation they faced in the clinical setting in many cases:

"We are told in the class to start oxytocin [for labor induction] from 4 drops... then in the hospital, they [midwives] start it from 20 drops...Well, why should that be like this? Which one should we learn then?" (Semester 5 student)

Another difficulty in this area was raised from the lack of continuity of the clinical instructors. Sometimes, one practical study unit in a clinical placement was educated by different instructors each week. Each of these trainers had their own specific methods and expectations in the procedures. As a result, the students became confused and unsecured:

"I have managed eight births until now, during which every trainer sutured an epi [episiotomy] in their own way ... Now I want to go to semester 8, but I still do not know how to suture an epi [episiotomy]." (Semester 7 student)

### Stress in the clinical setting

The students mentioned several conditions that made them stressed. Most of them expressed fear that they might have done something wrong or doing harm to the mother and baby:

“One fear that occupied my mind was that the baby might fall from my hands during the birth… In those days [first year] I was not confident enough and was really scared of these types of concerns.” (Semester 6 student)

Also, some participants reported heavy stress in their first birth experience:

"The first time I was at a birth, I was afraid a lot. It was terrible. The midwife cut the perineum… I was so scared… This was terrifying… The scissor was tearing the tissue apart… It was scary to tear apart someone’s muscles… And then, the birth of the baby’s head was terrifying too. His hair was stained with blood …" (Semester 6 student)

Inadequate facilities for the students in clinical placements were another source of stress for the students:

"In some clinical settings, there is not even a closet to put our stuff in… We do not have a resting place in some hospitals ... After a lot of work and fatigue in a busy day, there is not even a chair to sit on for a few minutes." (Semester 3 student)

Moreover, the human environment of clinical placement could be a source of stress for the students. Poor relationship of some clinical trainers and staff with students, struggling over birth management with residents, and fear of being reprimanded due to probable errors, were some of these types of stress sources:

"On the way to the hospital, I am always worried about lots of things…. Not to do anything wrong… Not being criticized by my instructor or midwives… How should be today’s fight [smile] over the birth … these are my real concerns which make me sick." (Semester 7 student)

## Discussion

This study set out with the aim of exploring the midwifery student experience of learning a clinical skill in Iran. The present findings seem to be consistent with other studies in Iran ^14^^,^^15^^,^^24^ which found the mismatch between the number of students in training groups and clinical training resources was problematic for midwifery students. Sending students to extra non-educational clinical placements without support of a clinical trainer in order to achieve the 80 required births, could not be a good solution. Indeed, as according to the International Confederation of Midwifery (ICM), the presence of clinical trainer in clinical placements is essential for midwifery learning.^1^ Like that was identified in Blåka’s^25^ study, absence of trainer not only imposed significant stress on our participants, but also deprived them from receiving feedback from the trainers that is essential for clinical education. This decreased the clinical learning outcomes for the participants.

The students stated that when coming back to the ward after a time gap during semester six, they had forgotten a lot of what they had learned before. A similar finding is also shown in the study of Van Dalen and colleagues^26^ in which they demonstrated skills that were learned by the students declined if not maintained by practice.

The students had difficulty with the simultaneity of theoretical and practical units that resulted in entering the clinical setting before becoming familiar with relevant theoretical lessons. This problem was raised especially at the beginning of semesters, and negatively influenced the quality of clinical education of participants. It is obvious that psychomotor learning must be obtained by having related cognitive skills. This principle has been articulated in the literature.^27^

The participants strongly emphasized the need for receiving support from their clinical instructors and staff. This is an expected finding because according to Cohen’s socialization model of novice students, the characteristics of students in the first stage are dependence and need for support.^28^ In our study, when the instructors behaved in a supportive manner, the learning of the students was facilitated. Conversely, when they imposed stress on the students or interfered too rapidly in a situation, their learning was inhibited.

A number of participants believed that some of their clinical instructors couldn’t express supportive behavior because they had low confidence in clinical situations. There are some possible explanations in this regard. Firstly, as described previously in this paper, clinical instructors in Iran are employees of the universities and are not hospital midwives. Some of them are academic individuals without having the experience of working in the clinical practice. As a result, they do not have enough confidence to carry out clinical jobs simultaneously as well as supporting students. Bringing midwifery teachers in the clinical area to work alongside students and staff has been regarded as an advisable approach in the literature. Bridging the theory-practice gap, teaching the care of women to students in a more realistic approach, and providing a more accurate assessment of students, are among the advantages that have been described in this regard.^7^^,^^29^ However, as articulated in the ICM education standards, it is essential that universities use experienced midwives for this objective.^1^ Moreover, maternity care in nearly all hospitals where students completed their placements was medical-led and very restrictive of autonomous midwifery practice. This condition limited the power of clinical instructors so that students felt they are low in confidence. Other studies also showed physician’s dominance as the limitation of clinical training of midwifery trainers.^7^^,^^30^

There were numbers of motives that stimulated students for learning, including having interest in midwifery practice, trying to obtain the competency for passing the final exam, and working as a midwife afterward. Literature related to learning has divided learning incentives into internal and external factors. Accordingly, factors such as interest and curiosity are internal stimulators, and the need to enhance job skills is external stimulators.^31^^,^^32^ Similar to a study on medical students, both of these factors were found simultaneously in our participants.^32^ However, in adult learners, internal motivations are more expected,^31^ and it seems to be preferable since it does not impose stress on learners.

The students reported that when they were exposed to different methods of carrying out clinical procedures, they became confused. A condition that triggered confusion between different methods in our participants was the gap between theory and practice. To facilitate the development of skills in students, clinical environments should have the chance to view and translate theoretical knowledge.^33^ Such environments facilitate clinical learning.^8^ But as shown in various studies, observation of differences between procedures done in hospitals and academic teachings creates confusion,^34^^,^^35^ discouragement,^8^ stress, and even a desire to quit the educational program.^36^ Moreover students had enormous difficulty with different methods that were taught by different clinical educators. This made them confused and unsecured. According to the literature,^7^^,^^29^^,^^37^^,^^38^ novice students tend to harmony and security of learning by one method, and by obtaining more experience and confidence, they desire getting to know different methods of different trainers. Thus, junior students or those who have newly entered a new clinical ward will benefit from having a permanent trainer. Also, a single trainer will be better able to assess students' clinical development and organize useful clinical experience. In addition, one-to-one interactions usually facilitate a supportive relationship.

The participants described several sources of stress during their clinical placement that could be grouped in challenges arising from the acquisition of clinical skills, physical and human environment. Because of the complexity and challenge, stress is an indispensable part of midwifery clinical education.^37^ Many participants of Sidebotham’s^33^ study considered the nature of the clinical midwifery is setting hard and stressful. Students in Lave’s^39^ study described their first encounter with the maternity wards hard and felt vulnerable, despite having nursing experience before entering the midwifery training.

Stress of the first presence in the clinical setting, stress in learning new skills, and fear of hurting the patients which were discovered in our participants, have also been mentioned in other studies on medical sciences students.^40^^-^^42^ However, neither of these studies portray the heavy stress displayed by our students during their first birth experience. One explanation would be that the participants of this study were young, direct entry students who had no exposure to clinical environments before. These findings highlight the importance of appropriate and adequate preparation of students before entering the clinical setting.

Lack of facilities in clinical environments was a significant source of stress in participants. Given similar findings in numbers previous studies in Iran,^14^^-^^16^^,^^21^ it seems that improving the facilities and the physical environment of educational hospitals is a necessity.

Additionally, students mentioned the poor relationship between some of the instructors and staff with them was another source of stress. The damaging effects of negative learning environment have been explained in several studies.^33^^,^^43^^,^^44^ In Thunes’study,^45^ the most effective factor on the well-being of midwifery students was the relationship they had with their mentors. These findings reveal the importance of expanding communication skills for clinical trainers to reduce stress in students.

The inappropriate and sometimes hostile behavior of staff was also a major source of stress in our participants. Poor relationship of health care workers with students has been identified as a deterring factor for clinical training in several studies.^17^^,^^21^^,^^45^^-^^48^ It may be that the stressful nature of midwifery practice leads the midwives to behave in this manner. Studies in Iran indicate high levels of bullying, professional burnout and job dissatisfaction in health workers, including midwives.^49^^-^^52^ In such circumstances, mistreatment of students is not unexpected, because, as smartly explained by Condell, it is not surprising that staff who are bullied but are not able to take out their anger on their oppressors, become frustrated and then abuse the students with whom they work.^53^

Finally, a number of important limitations need to be considered. First, this study held only one focus group discussion. This may not yield an adequate range and depth of midwifery student experience of clinical skills training. However, the combination of individual interviews and focus group discussion contributed to reaching data saturation. A further limitation of the study is that some of the students were interviewed by their trainer. One may argue that if students were interviewed with someone else, we could explore different experiences of clinical skills training. Given these limitations, caution must be applied as findings might not be transferable to other settings.

## Conclusions

This study showed a number of difficulties and concerns that students had during their clinical learning. These included restricted clinical learning opportunities and facilities, stress in encountering their first birth, fear of doing harm, confusion between different methods, trouble in relationships with their instructors and staff, and receiving poor support. The findings of this study have a number of important implications for midwifery education and future practice. Universities are required to harmonize the number of students they admit in the educational program with their clinical capacities in a way that they ensure students’ access to learning opportunities. Also, it is suggested to review the midwifery course curriculum to determine if it is possible to provide the obstetrics study units continuously and without time gaps. Moreover, since the students enter the educational program without previous experience of hospitals, more emphasis should be put on preclinical preparation to help them transition to the clinical setting. So, exploration by the curriculum planners is required to determine whether it is viable to provide increased pre-clinical hours in skill labs to prepare the students better.

Empowering midwives in Iran would be beneficial, as they would become more satisfied with their profession and so would be more helpful in the education of newcomers.

Also, to establish optimal clinical environments for students learning midwifery, staff should be trained to become more student-oriented. Universities should provide programs for expanding the clinical instructors’ competencies in terms of educational and clinical skills. Furthermore, it is desirable to provide consistent instructors for clinical education of novice students to reduce their confusion because of seeing different ways of doing things. Moreover, the methods of performing procedures should be unified between different clinical trainers by periodic coordination programs. Finally, we suggest that a similar study to this one should be carried out in other universities to gain a better insight of the current status of midwifery education in Iran.

### Acknowledgements

Isfahan University of Medical Sciences, Isfahan, Iran, supported this study financially. We would like to thank the midwifery students who volunteered to participate in this study. The creation of this study would have been impossible without their contribution.

### Conflicts of Interest

The authors declare that they have no conflict of interest.

## References

[1] ICM. International confederation of midwives, global standards for midwifery education. [Cited 18 February 2017]; Available from http://www.internationalmidwives.org. 10.1016/j.midw.2011.04.001 21550149

[2] Fullerton JT, Thompson JB (2005). Examining the evidence for The International Confederation of Midwives' essential competencies for midwifery practice.. Midwifery.

[3] Nicol M, Freeth D (1998). Assessment of clinical skills: a new approach to an old problem.. Nurse Educ Today.

[4] McIntosh T, Fraser DM, Stephen N, Avis M (2013). Final year students' perceptions of learning to be a midwife in six British universities.. Nurse Educ Today.

[5] Lake S, McInnes RJ (2012). Exploring cognitive skill development in midwifery education.. Nurse Educ Pract.

[6] Armstrong N (2008). Role modelling in the clinical workplace.. British Journal of Midwifery.

[7] Licqurish S, Seibold C (2008). Bachelor of Midwifery students' experiences of achieving competencies: the role of the midwife preceptor.. Midwifery.

[8] Longworth MK (2013). An exploration of the perceived factors that affect the learning and transfer of skills taught to student midwives.. Midwifery.

[9] Khajehei M, Ziyadlou S, Hadzic M, Kashefi F (2011). The genesis and consequences of stress among midwifery students.. British Journal of Midwifery.

[10] Ehsanpour S. Achieving minimum learning requirements from the viewpoints of midwifery students in Isfahan school of nursing and midwifery. Iranian Journal of Medical Education. 2006;6:17-24.

[11] Delaram M, Safdari Dahcheshme F, Banayian S, Kazemian A, Sereshti M, Raeisi Z, et al. Midwifery students' self assessment of their ability in practical skills. Quarterly of Education Strategies in Medical Sciences. 2013;6:177-182.

[12] Mousavi P, Montazeri S. Evaluating the achieving rate of learning minimums in clinic and gynecology units and their performance obstacles from midwifery students' viewpoint of nursing and midwifery school in Ahvaz. Educational Development of Jundishapur. 2013;3:71-80.

[13] Beigmoradi A, Nazeri H. Evaluation of achievement to curriculum objectives in nursing, midwifery and operating room technician students of Hamedan and Shiraz Universities of Medical Sciences. Iranian Journal of Medical Education. 2002;2:25-30.

[14] Homayounfar N, Mostafazadeh F, Asadzadeh F, Rostamnejhad M, Nazemi A. Clinical education from nursing and midwifery students' point of view, Ardebil University of Medical Sciences. In: First national Kongress on Nursing and Midwifery Clinical Education. Iran; 2009.

[15] Baraz Pordanjani S, Fereidooni Moghadam M, Loorizade M. Clinical education status according to the nursing and midwifery students' point of view, Tehran University of Medical Sciences. Strides in Development of Medical Education Journal of Medical Education Development Center. 2008;5:102-112.

[16] Omidvar S, Bakouee F, Salmalian H. Clinical education problems: the viewpoints of midwifery students in Babol Medical University. Iranian Journal of Medical Education. 2006;5:15-20.

[17] Ziaee S. Clinical education stressors from the view point of midwifery students of Islamic Azad University, Kazerun branch. Development Strategies in Medical Education. 2014;1:29-39.

[18] Pournamdar Z, Salehiniya H, Shahrakipoor M, Sohrabzade S. Nurse and midwifery students' satisfaction of clinical education in hospitals of Zahedan. Survey in medical sciences education. 2015;8:45-51.

[19] Haghani F, Rahimi M, Ehsanpour S. An investigation of "perceived feedback" in clinical education of midwifery students in Isfahan University of Medical Sciences. Iranian Journal of Medical Education. 2014;14:571-581.

[20] Pakpour V, Rahmani A, Salimi S, Shahim A. The viewpoint of midwifery students on support and supervision in clinical learning environment at Zanjan University of Medical Sciences. Educational Developement of Jundishapur. 2014;5:374-382.

[21] Shahoei R, Hesami K, Zaheri F,Hashemi Nasab L. The experience of graduated midwifery students about clinical education: a phenomenological study. Journal of Medical Educational and Development Yazd University of Medical Sciences. 2013;8:2-13.

[22] Gillham B. Research Interviewing: the range of techniques: a practical guide. London: McGraw-Hill Education; 2005.

[23] Graneheim UH, Lundman B (2004). Qualitative content analysis in nursing research: concepts, procedures and measures to achieve trustworthiness.. Nurse Educ Today.

[24] Yousefy A, Yazdannik Ar, Mohammadi S (2015). Exploring the environment of clinical baccalaureate nursing students' education in Iran; A qualitative descriptive study.. Nurse Educ Today.

[25] Blaka G (2006). Newcomers' learning of midwifery practice in a labour ward: a socio-cultural perspective.. Learn Health Soc Care.

[26] van Dalen J, Kerkhofs E, van Knippenberg-Van Den Berg BW, van Den Hout HA, Scherpbier AJ, van der Vleuten CP (2002). Longitudinal and concentrated communication skills programmes: two dutch medical schools compared.. Adv Health Sci Educ Theory Pract.

[27] Persson EK, Kvist LJ, Ekelin M (2015). Analysis of midwifery students' written reflections to evaluate progression in learning during clinical practice at birthing units.. Nurse Educ Pract.

[28] Chitty KK, Black BP. Professional nursing: concepts and challenges. Philadelphia: Elsevier; 2011.

[29] Begley CM (1999). A study of student midwives' experiences during their two-year education programme.. Midwifery.

[30] Baird K (2007). Exploring autonomy in education: preparing student midwives.. British Journal of Midwifery.

[31] Merriam SB. Andragogy and self-directed learning: pillars of adult learning theory. New Directions for Adult and Continuing Education. 2001;2001:3-14.

[32] Misch DA (2002). Andragogy and medical education: are medical students internally motivated to learn?. Adv Health Sci Educ Theory Pract.

[33] Sidebotham M, Fenwick J, Carter A, Gamble J. Using the five senses of success framework to understand the experiences of midwifery students enroled in an undergraduate degree program. Midwifery. 2015;31:201-207. 10.1016/j.midw.2014.08.007 25277735

[34] Morgan R (2006). Using clinical skills laboratories to promote theory-practice integration during first practice placement: an Irish perspective.. J Clin Nurs.

[35] Shin KR. The meaning of the clinical learning experience of Korean nursing students. J Nurs Educ. 2000;39:259-265.

[36] Carolan M (2011). The good midwife: commencing students' views.. Midwifery.

[37] Brunstad A, Giske T, Hjalmhult E. How midwifery students experience learning conditions in labor wards. J Nurs Educ Pract. 2016;6:136.

[38] Raisler J, O'Grady M, Lori J (2003). Clinical teaching and learning in midwifery and women's health.. J Midwifery Womens Health.

[39] Lave J, Wenger E. Situated learning: legitimate peripheral participation. New York: Cambridge University Press; 1991.

[40] Hart G, Rotem A (1994). The best and the worst: students' experiences of clinical education.. Aust J Adv Nurs.

[41] Pugh CM, Obadina ET, Aidoo KA (2009). Fear of causing harm: use of mannequin-based simulation to decrease student anxiety prior to interacting with female teaching associates.. Teach Learn Med.

[42] Sarikaya O, Civaner M, Kalaca S (2006). The anxieties of medical students related to clinical training.. Int J Clin Pract.

[43] Randle J (2001). The effect of a 3-year pre-registration training course on students' self-esteem.. J Clin Nurs.

[44] Obeidi N, Motamed N. Comparison of Students' and teachers' viewpoints about clinical education environment: a study in paramedical and nursing & midwifery schools of Bushehr University of Medical Sciences. Strides in Development of Medical Education Journal of Medical Education Development Center. 2011;8:88-93.

[45] Thunes S, Sekse RJ (2015). Midwifery students first encounter with the maternity ward.. Nurse Educ Pract.

[46] Begley CM (2002). 'Great fleas have little fleas': Irish student midwives' views of the hierarchy in midwifery.. J Adv Nurs.

[47] Khadivzadeh T, Farrokhi F. Evaluation of strengths and weaknesses of clinical training from day and night courses students' viewpoints in Mashhad nursing and midwifery faculty. Iranian Journal of Medical Education. 2003;3:67-68.

[48] Papp I, Markkanen M, von Bonsdorff M (2003). Clinical environment as a learning environment: student nurses' perceptions concerning clinical learning experiences.. Nurse Educ Today.

[49] Sotudeh Asl N, Bakhtiari A. Evaluation of occupational exhaustion and its related factors in nurses and midwives working in Semnan University of Medical Sciences. Journal of Kordestan Medical Sciences. 2006;11:77-83.

[50] Sabooteh S, Shahnazi H, Sharifirad G, Hassanzadeh A. The survey of job satisfaction of midwives working in labor wards of Isfahan hospitals. Journal of Health System Research. 2014;10:191-200.

[51] Mirmolaei T, Dargahi H, Kazemnejad A, Mohajerrabhari M. Job satisfaction of midwives. Hayat, The Journal of Faculty of Nursing & Midwifery. 2005;11:87-95.

[52] Shakerinejhad M. Evaluation of satisfaction of different job factors in midwives working in labor and delivery rooms and its relation with their demographic characteristics in hospitals of Tehran. In: Midwifery. Iran: Iran University of Medical Sciences; 1994.

[53] Condell SL. Nurse bullying-a preliminary analysis. In: Women's studies University College Dublin; 1995.

